# Supplementary material for: Peer Support and Mental Health of Migrant Domestic Workers: A Scoping Review
Source: Int J Environ Res Public Health. 2022 Jun 22;19(13):7617. doi: 10.3390/ijerph19137617 (PMC9265321; doi:10.3390/ijerph19137617)
Supplement: Supplementary file 1 [file ijerph-19-07617-s001.zip › ijerph-1728914-supplementary.pdf]

File S1 Example search strategy for OVID Medline

[1] exp migrant worker/

[2] ((migra* or immigra* or foreign* or non-native or transient* or economic) adj2 (work* or labor* or labour*)).ti,ab,hw.

[3] (domestic helper* or domestic worker*). ti,ab,hw.

[4] or/1-3

[5] exp peer group/

[6] exp social support/

[7] exp self-help group/

[8] peer*. ti,ab,hw.

[9] (lay* adj2 (led or lead* or run* or conduct* or person* or people* or work* or provider* or advisor* or consultant* or educator* or train* or tutor* or instructor* or facilitator*)).ti,ab,hw.

[10] (mutual* adj2 (aid* or support* or help*)).ti,ab,hw.

[11] or/5-10

[12] 4 and 11

[13] limit 12 to (humans and yr="2001 - 2021")
